# Risk Factors for Neonatal Sepsis: A Retrospective Case-Control Study among Neonates Who Were Delivered by Caesarean Section at the Trauma and Specialist Hospital, Winneba, Ghana

**DOI:** 10.1155/2018/6153501

**Published:** 2018-12-19

**Authors:** Peter Adatara, Agani Afaya, Solomon Mohammed Salia, Richard Adongo Afaya, Anthony K. Kuug, Ethel Agbinku, Eric Agyabeng-Fandoh

**Affiliations:** ^1^School of Nursing and Midwifery, University of Health and Allied Sciences, Ho, Ghana; ^2^Department of Nursing, Kwame Nkrumah University of Science and Technology, Kumasi, Ghana

## Abstract

The third Sustainable Development Goal (SDG) for child health, which targets ending preventable deaths of neonates and children under five years of age by 2030, may not be met without substantial reduction of neonatal sepsis-specific mortality in developing countries. This study aimed at assessing the prevalence and risk factors for neonatal sepsis among neonates who were delivered via caesarean section. A retrospective case-control study was conducted among neonates who were delivered via caesarean section at the Trauma and Specialist Hospital, Winneba, Ghana. Data collection lasted for 4 weeks. The extracted data were double-entered using Epidata software version 3.1 to address discrepancies of data entry. Descriptive statistics such as frequencies and percentages of neonatal characteristics were generated from the data. Both univariate and multivariate logistic regression were used to determine associations between neonatal sepsis and neonatal characteristics with odds ratios, 95% confidence intervals, and p values calculated using variables that showed significant association (p<0.05) in the chi-square analysis for the multivariate logistic regression. A total of 383 neonates were recruited; 67 (17.5%) had sepsis (cases). The neonatal risk factors associated with sepsis were birth weight (*χ*2=6.64, p=0.036), neonatal age (*χ*2=38.31, p<0.001), meconium passed (*χ*2=12.95, p<0.001), reason for CS (*χ*2=24.27, p<0.001), and the duration of stay on admission (*χ*2=36.69, p<0.001). Neonatal sepsis poses a serious threat to the survival of the newborn as the current study uncovered 6.0% deaths among sepsis cases. The findings of this study highlight the need for routine assessment of neonates in order to identify risk factors for neonatal sepsis and to curb the disease burden on neonatal mortality.

## 1. Introduction

Since 1985 the ideal rate for cesarean section (CS) proposed by the International Healthcare Community is supposed to be between 10% and 15%. However, CS delivery has seen a great increase among developed and developing countries [1, 2]. In recent times clinicians and governments have all over the world expressed a greater concern about the upsurge of CS deliveries and its potential risk for complications to the mother and the neonates' health, if surgical intervention is not medically justified but based on maternal request [3]. Evidence shows that maternal mortality and morbidity can effectively be prevented when medical justification for CS is recommended [3]. Recent systematic review and ecological analysis done on CS deliveries showed that CS rates above 10% were not related to the reduction of maternal and neonatal mortality rates [4, 5]. Thapa and colleagues in their study in Kathmandu, Nepal, revealed that deliveries via CS are a predictor of neonatal sepsis [6]. Neonates delivered by cesarean section might probably be at risk for laceration from sharp instruments during the procedure. Fetal laceration occurs in about 0.1% to 3.1% of CS deliveries [7, 8] and this can be a possible route of entry of microorganisms leading to neonatal sepsis.

The third Sustainable Development Goal for child health (United Nations 2015), which aims to end preventable deaths of neonates and children under five years of age by 2030, may not be met without substantial reduction of neonatal sepsis-specific mortality in the developing countries [9]. Early identification of neonatal risk factors for sepsis would enable early clinical diagnosis and treatment aiming to reduce morbidity and mortality. Although previous studies elsewhere in the developed countries, as highlighted above, have examined and identified some risk factors for neonatal sepsis, there is limited evidence on neonatal risk factors for sepsis among CS deliveries in developing countries, particularly Ghana. This study aimed at assessing the prevalence and risk factors for neonatal sepsis among neonates who were delivered via caesarean section.

## 2. Methodology

### 2.1. Research Design

A retrospective case control study was conducted among neonates who were delivered by caesarean section in the Trauma and Specialist Hospital, Winneba, Ghana. Neonatal sepsis was diagnosed based on laboratory investigations and Integrated Management of Neonatal and Childhood Illness (IMNCI) clinical features.

### 2.2. Study Setting

The study was conducted at the Trauma and Specialist Hospital, Winneba, Ghana. The Trauma and Specialist Hospital currently serves as the Central Regional Hospital. The paediatric ward doubles as the Neonatal Intensive Care Unit (NICU) with 4 incubators and 5 phototherapy machines. The NICU has two cubicles/wards designated for only neonatal cases, 5 beds and 4 cots.

### 2.3. Data Collection

Data collection started after ethical approval and permission sought from the facility. Four (4) research assistants were recruited and trained by the principal investigator about the main aim of the study and how to extract information from neonatal medical records in the study tool/checklist. The neonatal medical identification record numbers were sent to the Records Department (RD) for folder retrieval (medical records). Four records personnel were involved in folder search and retrieval from the RD. About 30-40 neonatal folders were retrieved from the RD each day. Caesarean section deliveries that were not from the Trauma and Specialist Hospital were exempted from the study. Data collection lasted for 4 weeks and 650 medical records (folders) were retrieved; 383 neonatal medical records were considered valid for the study, out of which 67 were considered cases while 316 were considered as controls.

### 2.4. Data Collection Tool

A semistructured data collection tool was developed based on reviewed related literature on risk factors of neonatal sepsis [10, 11]. The data collection tool consisted of sociodemographic and neonatal characteristics (neonatal age on admission, sex, gestational age, birth weight, Apgar score at first and fifth minute, crying immediately at birth, resuscitation at birth, and duration of stay on admission).

### 2.5. Validity and Reliability

Information validity of the questionnaire sheet was determined through an extensive review of literature about risk factors of neonatal sepsis [10, 11]. The data collection tool was also face-validated by a midwife, paediatrician, and a gynaecologist. Reliability analysis was used to determine the extent to which the items in the questionnaire are related to each other. The pretest was conducted among 2016 admissions folders (5 cases and 20 controls) of the neonates. Results of the pilot study were also used to confirm reliability (test-retest reliability). The findings from validity and reliability suggested that the current data abstraction tool was a viable tool for data collection in this study.

### 2.6. Data Analysis

The extracted data was double-entered using Epidata software version 3.1 to address discrepancies of data entry. The data was then exported into STATA 14.0 for data cleaning and analysis. Descriptive statistics such as frequencies and percentages of neonatal characteristics were generated. Neonatal characteristics were then cross-tabulated with sepsis and associations were tested using chi-square tests and p value of less than 0.05 significance level was considered as statistically significant. Both univariate and multivariate logistic regression were used to determine associations between neonatal sepsis and neonatal characteristics with odds ratios, 95% confidence intervals, and p values calculated using variables that showed significant association (p<0.05) in the chi-square analysis for the multivariate logistic regression. Tables were used to present the results.

### 2.7. Ethical Consideration

Ethical approval for the study was obtained from the University of Health and Allied Sciences Research Ethics Committee (UHAS-REC/A.3 [8] 17-18). Data abstraction commenced after permission was granted by Trauma and Specialist Hospital management.

## 3. Results

### 3.1. Neonatal Characteristics

The current study documents a total of 67 neonates with sepsis (cases) and 316 neonates without sepsis (controls) who were delivered through caesarean section from January to December 2017. Majority of the neonates (cases) were below 7 days of age representing 55 (82.1%) while most of the neonates (controls) were between 7 and 28 days of age 188 (59.5%). Majority of the cases 34 (50.8%) were males while majority of the controls 163 (51.6%) were females. A higher proportion of the neonates had their birth weight greater than 2.5kg among cases 49 (73.2%) and among controls 235 (74.4%). Forty-nine (73.1%) cases and 258 (81.6%) were delivered at term through caesarean section. A higher proportion of neonates who had their APGAR score greater than seven in the 5^th^ minute were found in cases 50 (74.6%) than those who had their APGAR score greater than seven in the 1^st^ minute among cases 40 (59.7%) (Table 1).

### 3.2. Association between Neonatal Characteristics and Neonatal Sepsis

Table 1 shows that birth weight and neonatal age had a statistically significant association with neonatal sepsis (*χ*2=6.64, p=0.036 and *χ*2=38.31, p<0.001, respectively). There was also significant association between the passing of meconium and neonatal sepsis (*χ*2=12.95, p<0.001). Furthermore, the reason for having a caesarean section and the duration of stay on admission also had a significant association with neonatal sepsis (*χ*2=24.27, p<0.001 and *χ*2=36.69, p<0.001, respectively).

### 3.3. Association between Neonatal Characteristics and the Odds of Neonatal Sepsis (Unadjusted)

Neonatal factors that influenced the occurrence of sepsis also showed that neonates with birth weight less than 1.5Kg were 2 and a half times more likely to have neonatal sepsis as compared to those with normal birth weight, greater than 2.5Kg [COR=2.54 (95%CI: 1.07, 6.03), p=0.035]. Neonates who were less than 7 days were 6.7 times more likely to have sepsis as compared to those who were 7 days and above [COR=6.73 (95%CI: 3.47, 13.07), p<0.001]. Neonates who also passed meconium were 3.8 times more likely to have sepsis as compared to those who passed none [COR=3.83 (95%CI: 1.77, 8.30), p=0.001] and [COR=8.30 (95%CI: 3.66, 18.61), p<0.001], respectively. Those among whom elective caesarean section was performed were 85% less likely to have neonatal sepsis as compared to those with emergency caesarean section and neonates who stayed on admission for greater than 2 weeks were 16.6 times more likely to have sepsis as compared to those who stayed for less than one week [COR=0.15 (95%CI: 0.07, 0.36), p<0.001] and [COR=16.6 (95%CI: 5.08, 54.31), p<0.001], respectively (Table 2).

### 3.4. Association between Neonatal Characteristics and the Odds of Neonatal Sepsis (Adjusted)

Neonates younger than 7 days of age were 9.4 times more likely to have sepsis as compared to those who were or were older than 7 days of age [AOR=9.40 (95%CI: 4.24, 20.81), p<0.001]. Respondents who had elective caesarean section were 86% less likely to have sepsis among neonates as compared to those who had emergency caesarean section [AOR=0.14 (95%CI: 0.05, 0.36), p<0.001]. More so, those who stayed on admission for a period greater than 2 weeks were 12 times more likely to have neonatal sepsis than those who stayed for less than a week [AOR=12.05 (95%CI: 2.56, 56.78), p=0.001] (Table 2).

### 3.5. Neonatal Risk Factors That Influence Early Neonatal Sepsis

Table 3 shows that there was a significant association between birth weight and early onset of neonatal sepsis (EONS) (*χ*2=6.96, p=0.031). APGAR scores in the first and fifth minute both showed a significant association with EONS (*χ*2=8.32, p=0.004) and (*χ*2=5.22, p=0.022), respectively. Neonates passing out meconium had a significant association with EONS (*χ*2=13.77, p<0.001). Early onset of neonatal sepsis was again significantly associated with reason for caesarean section and duration of stay on admission (*χ*2=18.57, p<0.001) and (*χ*2=28.99, p<0.001), respectively.

Logistic regression analysis revealed that neonates whose birth weight was below 1.5Kg were almost 4 times more likely to have EONS as compared to those with normal birth weight greater than 2.5Kg [COR=3.96 (95%CI: 1.22, 12.87) p=0.022]. Also, neonates whose APGAR score in the first and fifth minute was less than 7 were 2.69 and 2.45 times more likely to have EOS neonatal sepsis as compared to those whose APGAR scores were greater than or equal to 7 [COR=2.69 (95%CI: 1.36, 5.33) p=0.005] and [COR=2.45 (95%CI: 1.12, 5.36) p=0.025], respectively. Those who had elective caesarean section were 83% less likely to have EOS of neonatal sepsis as compared to those who had emergency caesarean section [COR=0.17 (95%CI:0.07, 0.41) p<0.001]. Neonates that stayed on admission for the periods 1 to 2 weeks and over two weeks were 5.28 times and 33.97 times more likely to have neonatal sepsis as compared to those who stayed on admission for less than one week [COR=5.28 (95%CI:1.62, 17.10) p=0.006] and [COR=33.97 (95%CI:4.09, 266.13) p=0.001], respectively.

Also neonates who were delivered through elective CS were 75% times less likely to have neonatal sepsis as compared to those delivered through emergency CS [AOR=0.25 (95%CI: 0.09, 0.68) p=0.006] and those who stayed on admission for over two weeks were 34.4 times more likely to have neonatal sepsis as compared to those who stayed for less than a week [AOR=34.44 (95%CI: 3.06, 388.02) p=0.004].

## 4. Discussion

Out of a cohort of 383 CS deliveries in the current study, 67 (17.5%) neonates had sepsis (cases). The prevalence of neonatal sepsis across the globe varies per country specific, the lowest found among developed countries as compared to that of developing countries having the highest prevalence. It is noted among some developing nations especially Africa and Asia to have neonatal sepsis incidence within 23-38/1,000 live births [12]. The current study shows an incidence of 17.5/1,000 live births which is lower than some African and Asian countries. The low incidence of neonatal sepsis among the study population as compared with some developing countries might be due to the hospital-based CS deliveries among mothers in the current study population.

This study found that birth weight, meconium passed, reason for CS, duration of stay at the facility, and neonatal age had significant association with neonatal sepsis. Further logistic regression analysis revealed that neonates who had their birth weight less than 1.5kg were 2.54 times more likely to be at risk of neonatal sepsis than those neonates who had normal birth weight. Similarly, a study by Dhumal et al. concluded that low birth weight (LBW) infants are at high risk of developing neonatal sepsis as compared to neonates with normal birth weight [13]. It was also observed that neonates that passed meconium intrauterine had 3.83 higher odds of developing neonatal sepsis than those neonates that did not pass meconium. Our finding is congruent with Siakwa et al., where they observed that women who had meconium stained liquor were 3.37 times more likely to have their neonates developing sepsis than women whose neonates did not pass meconium [10]. This might probably be due to the fact that neonates that passed meconium might have suffered asphyxia in utero and asphyxiated neonates are most likely to be resuscitated after CS birth. Though our study did not find any significant association between resuscitation and neonatal sepsis, a study by Siakwa et al. found infants who were resuscitated at birth to be 5.726 times more likely to develop neonatal sepsis compared to those who were not resuscitated [10]. It was interesting to note that neonates who stayed on admission for more than 2 weeks were 16.6 times more likely to have sepsis as compared to those who stayed for less than one week. This is possible because neonates that have longer duration of stay in the health facility would be predisposed to nosocomial infection due to their immature immune system.

The study observed that early onset of neonatal sepsis (EONS) was high among cases (82.1%). Birth weight, Apgar score in the first and fifth minute, passing out meconium, and duration of stay at the facility were strongly related to the risk of developing early neonatal sepsis. Neonates who had birth weight below 1.5Kg were almost 4 times more likely to have EONS as compared to those with normal birth weight >2.5Kg. The present study finding is congruent with Bhat and Baby who reported low birth weight as a risk factor of EONS. They also observed that neonates who had LBW had 10 times higher odds of developing early neonatal sepsis as compared to the normal birth weight. Our study also found that as birth weight decreases, more likely neonates will develop EONS. This present study finding is consistent with several other studies where they found Apgar score <7 in the first minute to be significantly related to EONS [14–16]. The study also revealed that low Apgar score <7 in the fifth minute was significantly associated with EONS. Some previous studies have also reported similar finding [17, 18]. Neonates with low Apgar score tend to have poor adaptation to extra uterine life due to the stress experienced during labour and therefore are more prone to infection.

### 4.1. Limitation of the Study

Since the study was retrospectively conducted on only admitted neonates born through CS in the hospital excluding those that were referred from other hospitals, thus the results might lack generalizability to the total population of sepsis cases recorded in the hospital.

## 5. Conclusion

The study identified birth weight, neonatal age, meconium passed, reason for CS, and the duration of stay on admission among CS deliveries as risk factors significantly associated with neonatal sepsis. Neonatal sepsis poses threat to the survival of the newborn as the current study uncovered 6.0% deaths among sepsis cases. The current findings highlight the need for routine assessment of neonates in order to identify risk factors for neonatal sepsis and to curb the disease burden on neonatal mortality. The study recommends further research on maternal risk factors for neonatal sepsis among CS deliveries in order to identify maternal factors associated with neonatal sepsis.

## Figures and Tables

**Table 1:** Association between neonatal characteristics and neonatal sepsis.

**Variable**	**Cases**	**Controls**	**Total**	**Chi-square, ** **χ** **2 (p-value)**
	**n=67(**%**)**	**n=316(**%**)**	**N=383(**%**)**	
**Gestational age (weeks)**				
<37	16 (23.9)	42 (13.3)	58 (15.1)	
37-42	49 (73.1)	258 (81.6)	307 (80.2)	5.10 (0.078)
>42	2 (3.0)	16 (5.1)	18 (4.7)	
**Birth weight (Kg)**				
<1.5	9 (13.4)	17 (5.4)	26 (6.8)	
1.5-2.5	9 (13.4)	64 (20.2)	73 (19.1)	6.64 (0.036)
>2.5	49 (73.2)	235 (74.4)	284 (74.1)	
**Sex**				
Male	34 (50.8)	153 (48.4)	187 (48.8)	
Female	33 (49.2)	163 (51.6)	196 (51.2)	0.12 (0.729)
**Neonatal age (Days)**				
<7	55 (82.1)	128 (40.5)	183 (47.8)	38.31 (<0.001)
≥7	12 (17.9)	188 (59.5)	200 (52.2)	
**APGAR score in the first minute**				
<7	27 (40.3)	96 (30.4)	123 (32.1)	2.49 (0.114)
≥7	40 (59.7)	220 (69.6)	260 (67.9)	
**APGAR score in the fifth minute**				
<7	17 (25.4)	58 (18.3)	75 (19.6)	1.73 (0.789)
≥7	50 (74.6)	258 (81.7)	308 (80.4)	
**Meconium**				
None	8 (11.9)	108 (34.2)	116 (30.3)	
Passed	59 (88.1)	208 (65.8)	267 (69.7)	12.95 (<0.001)
No	55 (82.1)	254 (80.4)	309 (80.7)	
Yes	12 (17.9)	62 (19.6)	74 (19.3)	0.10 (0.747)
**Reason for CS**				
Emergency	60 (89.5)	182 (57.6)	242 (63.2)	
Elective	7 (10.5)	134 (42.4)	141 (36.8)	24.27 (<0.001)
**Duration of stay**				
< 1 week	48 (71.6)	290 (91.8)	338 (88.3)	
1-2 weeks	8 (11.9)	22 (7.0)	30 (7.8)	
<2 weeks	11 (16.5)	4 (1.2)	15 (3.9)	36.69 (<0.001)
**Outcome of admission**				
Discharged	61 (91.0)	300 (94.9)	361 (94.3)	
Died	4 (6.0)	13 (4.1)	17 (4.4)	
Referred	2 (3.0)	3 (1.0)	5 (1.3)	2.27 (0.321)

**Table 2:** Neonatal factors that influence the occurrence of sepsis among neonates.

**Variable**	**Cases**	**Controls**	**Total**	**COR [95**%**CI] p-value**	**AOR [95**%**CI] p-value**
	**n=67(**%**)**	**n=316(**%**)**	**N=383(**%**)**		
**Gestational age (weeks)**					
<37	16 (23.9)	42 (13.3)	58 (15.1)	3.05 [0.62, 14.78] 0.167	
37-42	49 (73.1)	258 (81.6)	307 (80.2)	1.51 [0.34, 6.81] 0.585	
>42	2 (3.0)	16 (5.1)	18 (4.7)	Ref.	
**Birth weight (Kg)**					
<1.5	9 (13.4)	17 (5.4)	26 (6.8)	2.54 [1.07, 6.03] 0.035	0.82 [0.25, 2.65] 0.737
1.5-2.5	9 (13.4)	64 (20.2)	73 (19.1)	0.67 [0.31, 1.45] 0.311	0.54 [0.19, 1.50] 0.236
>2.5	49 (73.2)	235 (74.4)	284 (74.1)	Ref.	Ref.
**Sex**					
Male	34 (50.8)	153 (48.4)	187 (48.8)	Ref.	
Female	33 (49.2)	163 (51.6)	196 (51.2)	0.91 [0.54, 1.54] 0.729	
**Neonatal age (Days)**					
<7	55 (82.1)	128 (40.5)	183 (47.8)	6.73[3.47,13.07]<0.001	9.40[4.24,20.81]<0.001
≥7	12 (17.9)	188 (59.5)	200 (52.2)	Ref.	Ref.
**APGAR score in the first minute**					
<7	27 (40.3)	96 (30.4)	123 (32.1)	1.55 [0.90, 2.66] 0.116	
≥7	40 (59.7)	220 (69.6)	260 (67.9)	Ref.	
**APGAR score in the fifth minute**					
<7	17 (25.4)	58 (18.3)	75 (19.6)	1.51 [0.81, 2.81] 0.191	
≥7	50 (74.6)	258 (81.7)	308 (80.4)	Ref.	
**Meconium**					
None	8 (11.9)	108 (34.2)	116 (30.3)	Ref.	Ref.
Passed	59 (88.1)	208 (65.8)	267 (69.7)	3.83 [1.77, 8.30] 0.001	0.63 [0.14, 2.80] 0.542
**Resuscitated at birth**					
No	55 (82.1)	254 (80.4)	309 (80.7)	Ref.	
Yes	12 (17.9)	62 (19.6)	74 (19.3)	0.89 [0.45, 1.77] 0.748	
**Reason for CS**					
Emergency	60 (89.5)	182 (57.6)	242 (63.2)	Ref.	Ref.
Elective	7 (10.5)	134 (42.4)	141 (36.8)	0.15 [0.07, 0.36] <0.001	0.14 [0.05, 0.36] <0.001
**Duration of stay**					
< 1 week	48 (71.6)	290 (91.8)	338 (88.3)	Ref.	Ref.
1-2 weeks	8 (11.9)	22 (7.0)	30 (7.8)	2.20 [0.93, 5.22] 0.074	1.38 [0.48, 3.99] 0.551
<2 weeks	11 (16.5)	4 (1.2)	15 (3.9)	16.6 [5.08, 54.31] <0.001	12.05[2.56,56.78]0.001
**Outcome of admission**					
Discharged	61 (91.0)	300 (94.9)	361 (94.3)	Ref.	
Died	4 (6.0)	13 (4.1)	17 (4.4)	1.51 [0.48, 4.80] 0.482	
Referred	2 (3.0)	3 (1.0)	5 (1.3)	3.28 [0.54, 20.04] 0.199	

**Table 3:** Neonatal risk factors that influence early neonatal sepsis.

**Variable**	**Cases** **n=55(**%**)**	**Controls** **n=128(**%**)**	**Total** **N=183(**%**)**	**Chi-square, ** **χ** **2** **(p-value)**	**COR [95**%**CI]** **p-value**	**AOR [95**%**CI]** **p-value**
**Gestational age (weeks)**						
<37	13 (23.6)	18 (14.1)	31 (16.9)		2.17 [0.38, 12.49] 0.387	
37-42	40 (72.7)	104 (81.2)	144 (78.7)		1.15 [0.22, 5.96] 0.864	
>42	2 (3.6)	6 (4.7)	8 (4.4)	2.54 (0.282)	Ref.	
**Birth weight (Kg)**						
<1.5	8 (14.5)	5 (3.9)	13 (7.1)		3.96 [1.22, 12.87] 0.022	1.91 [0.46, 7.92] 0.372
1.5-2.5	9 (16.4)	29 (22.7)	38 (20.8)		0.77 [0.33, 1.77] 0.536	0.56 [0.19, 1.66] 0.298
>2.5	38 (69.1)	94 (73.4)	132 (72.1)	6.96 (0.031)	Ref.	Ref.
**Sex**						
Male	28 (50.9)	59 (46.1)	87 (47.5)		Ref.	
Female	27 (49.1)	69 (53.9)	96 (52.5)	0.36 (0.550)	0.82 [0.44, 1.55] 0.550	
**APGAR score in the first minute**						
<7	23 (41.8)	27 (21.1)	50 (27.3)		2.69 [1.36, 5.33] 0.005	1.40 [0.46, 4.27] 0.550
≥7	32 (58.2)	101 (78.9)	133 (72.7)	8.32 (0.004)	Ref.	Ref.
**APGAR score in the fifth minute**						
<7	15 (27.3)	17 (13.3)	32 (17.5)		2.45 [1.12, 5.36] 0.025	1.70 [0.46, 6.36] 0.426
≥7	40 (72.7)	111 (86.7)	151 (82.5)	5.22 (0.022)	Ref.	Ref.
**Meconium**						
None	8 (14.6)	55 (43.0)	63 (34.4)		Ref.	Ref.
Passed	47 (85.4)	73 (57.0)	120 (65.6)	13.77 (<0.001)	4.43 [1.94, 10.12] <0.001	0.82 [0.21, 3.18] 0.775
**Resuscitated at birth**						
No	45 (81.8)	108 (84.4)	153 (83.6)		Ref.	
Yes	10 (18.2)	20 (15.6)	30 (16.4)	0.18 (0.668)	1.20 [0.52, 2.77] 0.669	
**Reason for CS**						
Emergency	48 (87.3)	69 (53.9)	117 (63.9)		Ref.	Ref.
Elective	7 (12.7)	59 (46.1)	66 (36.1)	18.57 (<0.001)	0.17 [0.07, 0.41] <0.001	0.25 [0.09, 0.68] 0.006
**Duration of stay**						
< 1 week	37 (67.3)	122 (95.3)	159 (86.9)		Ref.	Ref.
1-2 weeks	8 (14.5)	5 (3.9)	13 (7.1)		5.28 [1.62, 17.10] 0.006	3.50 [0.96, 12.73] 0.057
<2 weeks	10 (18.2	1 (0.8)	11 (6.0)	28.99 (<0.001)	32.97 [4.09, 266.13] <0.001	34.44 [3.06, 388.02] 0.004
**Outcome of admission**						
Discharged	49 (89.1)	120 (93.7)	169 (92.4)		Ref.	
Died	4 (7.3)	7 (5.5)	11 (6.0)		1.40 [0.39, 4.99] 0.605	
Referred	2 (6.6)	1 (0.8)	3 (1.6)	2.21 (0.331)	4.90 [0.43,55.27] 0.199	

## Data Availability

The data used to support the findings of this study are available from the corresponding author upon request.
